# Anti-Freezing Conductive Ionic Hydrogel-Enabled Triboelectric Nanogenerators for Wearable Speech Recognition

**DOI:** 10.3390/ma18092014

**Published:** 2025-04-29

**Authors:** Tao Chen, Andeng Liu, Wentao Lei, Guoxu Wu, Jiajun Xiang, Yixin Dong, Yangyang Chen, Bingqi Chen, Meidan Ye, Jizhong Zhao, Wenxi Guo

**Affiliations:** 1 Department of Physics, College of Physical Science and Technology, Research Institution for Biomimetics and Soft Matter, Xiamen University, Xiamen 361005, China; 19820221153905@stu.xmu.edu.cn (T.C.); liuandeng@stu.xmu.edu.cn (A.L.); 19820221153856@stu.xmu.edu.cn (W.L.); guoxuwu_1@163.com (G.W.); xiangjiajun168@163.com (J.X.); dongyixin_edu@163.com (Y.D.); chenyy612@163.com (Y.C.); 17324918758@163.com (B.C.); mdye@xmu.edu.cn (M.Y.); 2 Beijing Institute of Nanoenergy and Nanosystems, Chinese Academy of Sciences, Beijing 101400, China; 3 School of Nanoscience and Technology, University of Chinese Academy of Sciences, Beijing 100049, China

**Keywords:** ionic conductive hydrogels, anti-freezing hydrogel, triboelectric nanogenerator, hydrogel-based TENGs, flexible sensors, speech recognition

## Abstract

Flexible wearable electronics face critical challenges in achieving reliable physiological monitoring, particularly due to the trade-off between sensitivity and durability in flexible electrodes, compounded by mechanical modulus mismatch with biological tissues. To address these limitations, we develop an anti-freezing ionic hydrogel through a chitosan/acrylamide/LiCl system engineered via the solution post-treatment strategy. The optimized hydrogel exhibits exceptional ionic conductivity (24.1 mS/cm at 25 °C) and excellent cryogenic tolerance. Leveraging these attributes, we construct a gel-based triboelectric nanogenerator (G-TENG) that demonstrates ultrahigh sensitivity (1.56 V/kPa) under low pressure. The device enables the precise capture of subtle vibrations at a frequency of 1088 Hz with a signal-to-noise ratio of 16.27 dB and demonstrates operational stability (>16,000 cycles), successfully differentiating complex physiological activities including swallowing, coughing, and phonation. Through machine learning-assisted analysis, the system achieves 96.56% recognition accuracy for five words and demonstrates good signal recognition ability in different ambient sound scenarios. This work provides a paradigm for designing environmentally adaptive wearable sensors through interfacial modulus engineering and ion transport optimization.

## 1. Introduction

With the rapid development of flexible electronics and the Internet of Things (IOT), flexible wearable electronic devices [1,2,3], as an emerging skin interface sensing technology, show great potential for application in the fields of health monitoring [4,5,6], human–computer interaction [7,8,9], and smart medicine [10,11,12]. Among them, triboelectric nanogenerators (TENGs), as an emerging energy harvesting technology, rely on the frictional electrification effect to collect mechanical energy from the environment and convert it into electrical signals without an external power supply, thus realizing a battery-free energy supply [13,14]. TENGs can detect very small forces and sense weak pressure, vibration, sound, touch, etc. It is very suitable for flexible electronic skin, health monitoring, touch sensing, and other fields.

Among various types of TENGs, those that use hydrogel as a flexible electrode material have attracted much attention due to the unique adjustable ionic conductivity advantage of hydrogel itself and the high transparency and flexibility of hydrogel electrodes, which are capable of adapting to a variety of deformation needs such as stretching, bending, folding, and pressing, so they show good application prospects in wearable self-energy devices [15,16,17,18,19]. However, hydrogels face some challenges in practical applications, such as narrow temperature range, poor stability, and insufficient environmental adaptability, which seriously limit their applications. Under high- and low-temperature environments, hydrogels tend to dry out or freeze, leading to performance degradation and affecting service life. Therefore, improving the temperature resistance of hydrogels is important for expanding the application of gel-based TENGs in diverse scenarios [20,21]. In recent years, the development of sustainable materials has received great attention, among which the development of bio-based materials is one of the key directions to promote sustainable development and green transformation [22,23]. As a naturally derived biopolymer, the construction of hydrogels based on chitosan not only provides a feasible solution for replacing traditional non-degradable materials but also opens up a new technological path to promote green and low-carbon development.

Here, by designing a chitosan hydrogel as an electrode, we constructed a hydrogel-based flexible G-TENG. The modulus of the hydrogel can be effectively adjusted by immersing it in different amounts of LiCl, which makes it more suitable for detection on the skin surface of the human body. In addition, the presence of LiCl endowed the hydrogel with higher ionic conductivity, good water retention, and anti-freezing ability. This makes this TENG soft with high detection sensitivity. We further fixed the G-TENG on the volunteer’s throat to realize the acquisition of sound signals, and through deep learning algorithms, we successfully realized speech recognition.

## 2. Materials and Methods

### 2.1. Materials

Chitosan (CS) was purchased from Aladdin Co., Ltd. (Shanghai, China). Acrylamide (AM), LiCl·H_2_O, tannic acid (TA), N,N′-methylene bisacrylamide (MBA), ammonium persulfate (APS), and acetic acid (CH_3_COOH) were all purchased from Macklin biochemical technology Co., Ltd. (Shanghai, China). FeCl_3_·6H_2_O was purchased from Xilong scientific Co., Ltd. (Shantou, China). All the chemicals were analytical grade and used without further purification.

### 2.2. Preparation of CS-PAM Hydrogel

CS-PAM Hydrogel is prepared according to the previous literature [24]. In brief, the AM solution was obtained by adding 2 g of acrylamide (AM), 4 mg of N,N′-methylene bisacrylamide (MBA), and 20 mg of ammonium persulfate (APS) to 3 mL of deionized water, followed by stirring at room temperature for 30 min until complete dissolution. Take 20 mL of chitosan solution (3%, dissolved in 1% acetic acid), add 20 mL of deionized water and 20 mg of tannic acid (TA), and stir for 30 min until complete dissolution. Immediately after that, 800 μL of ferric chloride hexahydrate (FeCl_3_·6H_2_O) at a concentration of 200 mg/mL was added to the above solution and stirred for 5 min to obtain the CS/TA/Fe solution. A total of 1 mL CS/TA/Fe solution was added to 1 mL of AM solution and then stirred for 30 s to obtain a uniform mixture. Next, the resultant solution was transferred to molds and put in a water bath at 60 °C. After 1 min, the PAM-CS hydrogels were achieved. The CS-PAM/LiCl hydrogels were achieved after immersing in LiCl aqueous solution with varying concentrations (1, 3, 5, and 8 M) for 20 min.

### 2.3. Manufacture of the G-TENG

The PDMS film was mixed with a Sylgard 184 base and curing agents (10:1 by weight). The CS-PAM hydrogel and PDMS were cut into the desired shape with a sharp blade. The final device was fabricated by wrapping and sealing the gel, PA, and PDMS with the elastomer films. A Cu wire was attached to the hydrogel as the electrical connection.

## 3. Results and Discussion

### 3.1. Preparation and Properties of the CS-PAM/LiCl Gel

**Figure 1:**
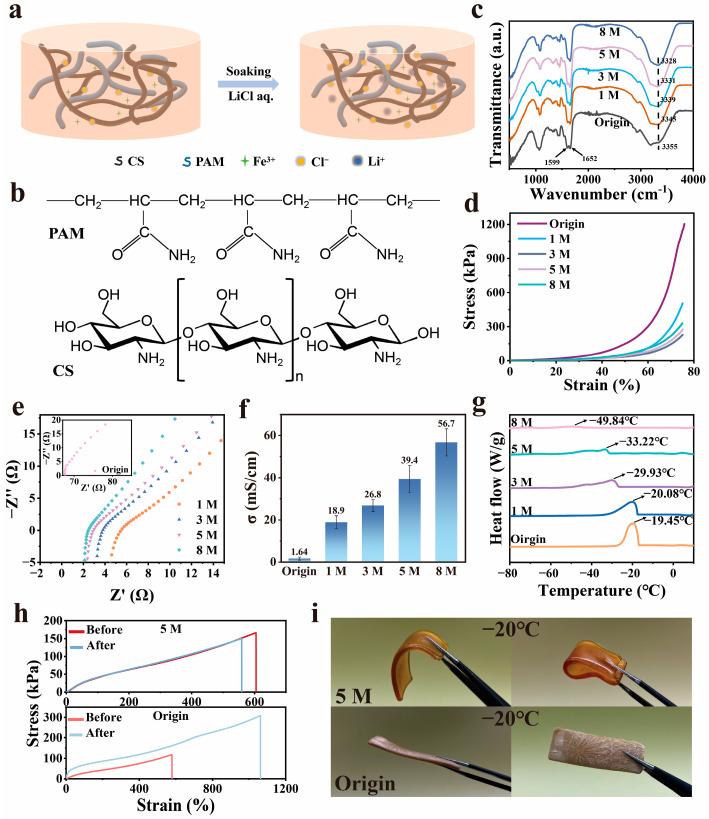
Characterization of the hydrogels. (**a**) Schematic illustration of the preparation of CS-PAM/LiCl hydrogels. (**b**) Polyacrylamide (PAM) macromolecules and chitosan (CS) macromolecules. (**c**) FTIR spectra of CS-PAM/LiCl hydrogels. (**d**) Compressive stress–strain curves of the hydrogels. Successive loading-unloading experiments in compression mode at 75% strain of the 0–8 M CS-PAM/LiCl hydrogels. (**e**) Nyquist plots of the hydrogels. (**f**) The ionic conductivities of the hydrogels. (**g**) DSC curves of the hydrogels. (**h**,**i**) Tensile stress–strain curves and optical images of original hydrogel and 5 M LiCl-treated hydrogel before and after freezing at −20 °C for 5 h.

In this paper, CS-PAM hydrogels were prepared by in situ polymerization of chitosan (CS) with acrylamide (AM) (Figure 1b). By introducing Fe^3+^ ions into the system and utilizing the abundant hydroxyl and amino groups on the CS molecular chain, combined with the multiple hydrogen bonds and metal–ligand bonds in the polymer network, a dynamic crosslinked network co-composed of CS, PAM, and Fe^3+^ was successfully constructed [24]. Subsequently, the resulting hydrogel was treated by immersing it in 1–8 M LiCl solution, during which Li^+^ and Cl^−^ ions gradually diffused into the interior of the hydrogel, and the salting-out effect prompted the entanglement of the CS-PAM molecular chains (Figure 1a) [25]. FTIR spectroscopy analysis showed that characteristic peaks were exhibited at 3355, 1652, and 1599 cm^−1^, corresponding to N-H and O-H bond stretching vibrations, C=O stretching (amide I), and N-H vibrations (amide II), respectively. After LiCl treatment, the shift in the characteristic peak at 3355 cm^−1^ to 3328 cm^−1^ and the increase in the intensity of the peak at 1652 cm^−1^ indicate that there is a further increase in the hydrogen-bonding interactions between aniline, hydroxyl, and amide groups within the hydrogel structure (Figure 1c). The prepared CS/PAM-LiCl hydrogels exhibited excellent compression deformation properties (Figure 1d), which can match the modulus of the skin better. We tested the ionic conductivity of the gels using stainless steel electrodes and the impedance model of the blocking electrode. By analyzing the Nyquist plots (Figure 1e) and the corresponding ionic conductivities (Figure 1f) of hydrogels treated with different concentrations of LiCl, it was found that the contact resistance between the CS-PAM hydrogels and the electrodes decreased significantly, and the ionic transport rate increased significantly with the increase in LiCl concentration. Among them, the hydrogel treated with 8 M LiCl exhibited the highest ionic conductivity of 56.7 mS cm^−1^. Based on the differential scanning calorimetry (DSC) analysis (Figure 1g), for the untreated hydrogel, an exothermic peak appeared at −19.45 °C, the exothermic peak continued to decrease with the increase in LiCl concentration, and the exothermic peak of the hydrogel treated with 8 M LiCl was −49.84 °C; the peak width was greater at this time, and the peak height was decreased, which indicated that the exothermic peak of hydrogel treated with 8 M LiCl could be effectively lowered by immersing in LiCl. This indicates that the solidification point of the hydrogel can be effectively reduced by soaking in LiCl. In order to evaluate the low-temperature performance of the hydrogel, the original gel and the 5 M LiCl-treated gel were placed in a −20 °C environment for 5 h, and the mechanical properties were tested immediately after thawing. The mechanical properties of both were examined by tensile testing (Figure 1h), and it was observed that the 5 M LiCl-treated gel maintained good tensile properties, with fracture strain rates of 604% and 558% before and after the low-temperature treatment, respectively, which showed a slight difference in the performance. In contrast, the tensile fracture rate of the untreated gel increased considerably, from 578% to 1059%, and exhibited plastic deformation behavior during stretching. From the optical images, it can be seen that the internal free water is completely frozen (Figure 1i), while the LiCl-treated gel still maintains good deformation properties and can be completely restored to its original state after bending, which indicates that the content of bound water in the treated gel is significantly increased [26].

### 3.2. Design of the G-TENG

**Figure 2:**
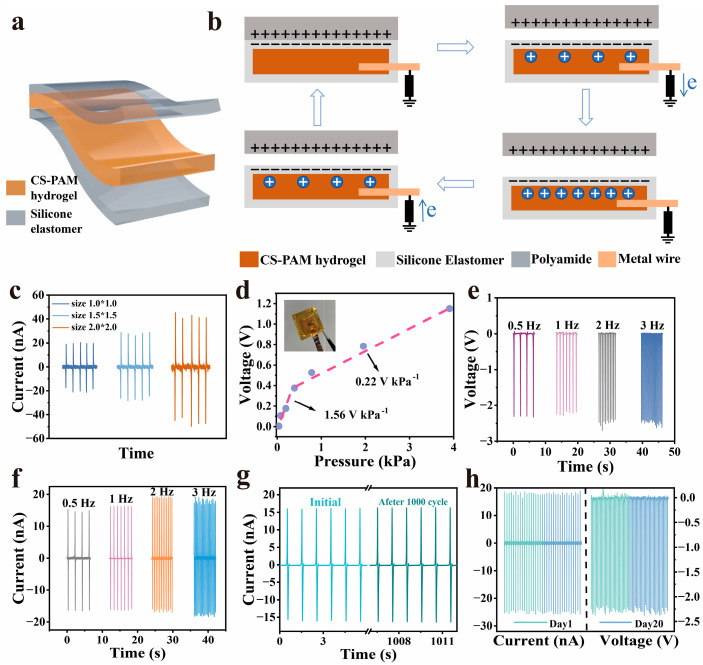
The work mechanism and electrical performance of the G-TENG. (**a**) Illustration of the hydrogel-based single-electrode TENG structure. (**b**) Working mechanism for electricity generation. (**c**) Short-circuit current of different sizes of the G-TENG. (**d**) Voltage sensitivity measurements of the G-TENG. (**e**,**f**) Frequency-dependent open-circuit voltage and short-circuit current of the G-TENG under 0.5–3 Hz. (**g**) Short-circuit current of the G-TENG before and after 1000 cycles. (**h**) Working repeatability and durability after 20 days.

Based on the excellent ion-conducting properties of CS-PAM/LiCl hydrogel, we apply it to the construction of a single-electrode friction nanogenerator (TENG), which realizes the conversion of mechanical energy to electrical energy and operates as a self-powered sensor. The conductive hydrogel prepared by post-treatment with 5 M LiCl solution was embedded in a polydimethylsiloxane (PDMS) friction layer as an electrode (Figure 2a), hereinafter referred to as a G-TENG. This TENG works based on the coupled mechanism of contact initiation and electrostatic induction in single-electrode mode (Figure 2b). Polyamide (PA) was used as the positive friction material and PDMS as the negative friction material in the experiments, and due to the difference in electronegativity between the two, when the two friction layers were in contact, the interfaces produced equal amounts of opposite-sign charges, respectively. During separation, ions in the hydrogel migrate under the influence of the electrostatic field on the PDMS surface, balancing the interfacial charges. This results in the formation of an excess ionic layer at the interface, which induces a double electric layer at the metal–hydrogel junction. Consequently, electrons flow through the copper conductor to the ground until electrostatic equilibrium is restored. Upon recontact, the process reverses: electrons return from the ground to the electrode, and the charge distribution resets to its initial state [27]. The output current of the G-TENG increases significantly with increasing device size (Figure 2c); based on this, a device with a size of 1.5 cm × 1.5 cm and a thickness of 1.5 mm was selected for system performance characterization in this study. Voltage response testing under varying pressures revealed a sensitivity of 1.56 V kPa^−1^ in the low-pressure range (0–0.39 kPa), which decreased to 0.22 V kPa^−1^ at higher pressures (0.39–3.90 kPa). The enhanced sensitivity at low pressures is attributed to the rapid expansion of the contact area between friction layers under applied loads, making the G-TENG particularly suitable for applications such as small vibration detection and low-intensity voice communication. Further investigation of the G-TENG’s output characteristics under different mechanical frequencies (Figure 2e,f) showed that both the voltage and current increase with frequency, peaking at 2.54 V and 18.9 nA (3 Hz). This improvement stems from accelerated interfacial charge transfer under high-frequency stimulation. To assess reliability, a 1000-cycle test at 1 Hz demonstrated stable performance, with the peak current maintained at 16.32 nA (Figure 2g). Additionally, aging tests over 20 days confirmed negligible degradation in the voltage and current output (Figure 2h), underscoring the device’s long-term operational stability.

### 3.3. Performance of G-TENG Sensors

**Figure 3:**
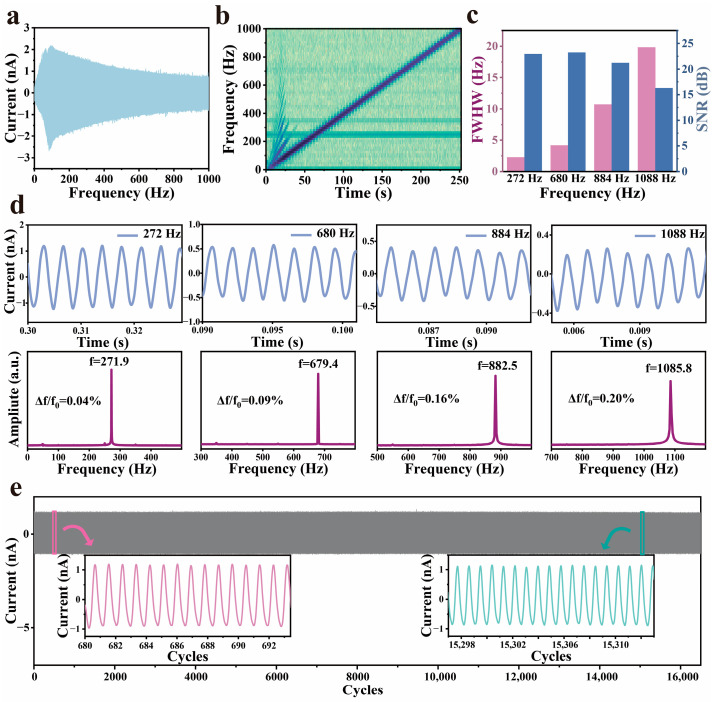
Performance of the G-TENG under tiny vibrations. (**a**) The amplitude-frequency curve of the G-TENG. (**b**) The frequency of the G-TENG response varies uniformly over time as the excitation signal. (**c**) FWHM and SNR. (**d**) The G-TENG response to different single-frequency excitations. (**e**) Stability testing of the G-TENG. The insets show the waveform at the beginning and the end, respectively.

To evaluate the small vibration detection capability of the G-TENG, the device was mounted on a vibration platform and subjected to a linearly swept sine wave excitation (0–1000 Hz) (Figure 3a). The results demonstrate high sensitivity in the sub-200 Hz frequency range, which aligns with the primary spectral components of human speech (50–200 Hz), confirming its suitability for vocal vibration detection. Time-domain analysis, in which the brightness of the color reflects the difference between large (dark) and small (bright) amplitudes, reveals a strong correlation between the G-TENG’s amplitude response and the excitation signal, with a uniform frequency detection rate of 4 Hz/s (Figure 3b). For quantitative frequency performance assessment, four characteristic frequencies (272 Hz, 680 Hz, 884 Hz, and 1088 Hz) representing distinct regions of the human voice spectrum were selected. Short-time Fourier transform (STFT) analysis (Figure 3d) shows excellent signal response after removing 50 Hz powerline interference and harmonics. Frequency relative error (Δf/f_0_) calculations indicate minimal deviations of 0.04%, 0.09%, 0.16%, and 0.20% at these frequencies, demonstrating high-fidelity vibration recording. Fast Fourier transform (FFT) analysis (Figure 3c) further reveals a frequency-dependent full width at half maximum (FWHM) values, reaching 18.81 Hz at 1088 Hz, highlighting the device’s superior frequency selectivity for discriminating single-frequency signals. Additionally, the G-TENG achieves a high signal-to-noise ratio (SNR) of 22.94 dB at 272 Hz, maintaining 16.27 dB even at 1088 Hz. Long-term stability tests involving 16,000 cycles at 34 Hz show no significant waveform degradation, with signal peaks retaining >99.2% of initial amplitude, underscoring exceptional operational durability.

### 3.4. Speech Recognition Function of G-TENG

**Figure 4:**
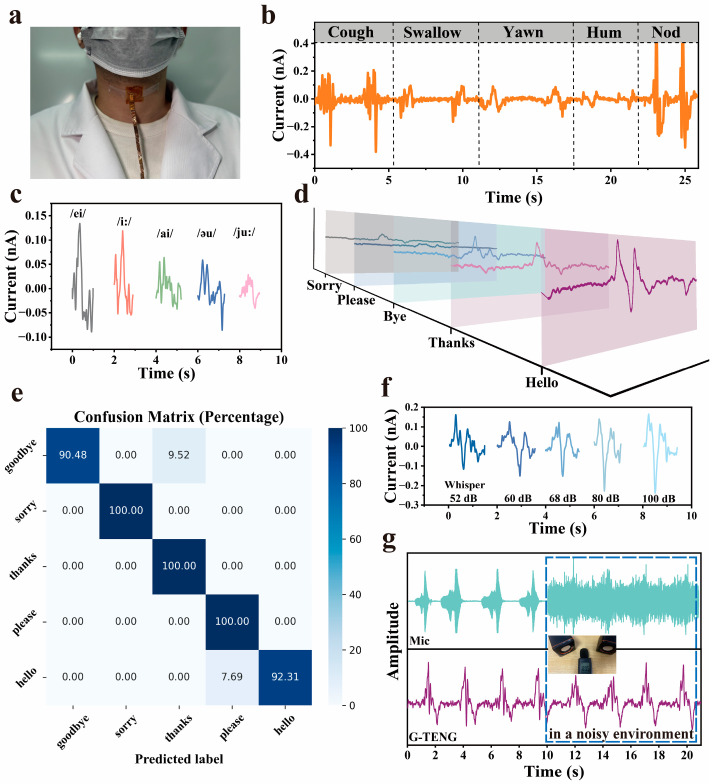
Acquisition of different movements of throat and speech signals. (**a**) Optical images of G-TENG. (**b**) Signal of different throat movements: coughing, swallowing, yawning, humming, and nodding. (**c**) Speech signal waveform of five vowels. (**d**) Speech signal waveform of 5 daily words. (**e**) Confusion matrix of 5 words. (**f**) Speech signal waveforms of the word “quiet” spoken at different volumes. (**g**) Comparisons of waveform changes using the G-TENG and a commercial microphone in quiet and noisy (80 dB) environments, respectively.

To assess the capability of G-TENG in capturing human throat vibrations, we mounted the device on a volunteer’s throat to systematically investigate its response to physiological activities (Figure 4a). The initial experiments recorded real-time current signals generated by common actions such as coughing and swallowing (Figure 4b). The results demonstrated the G-TENG’s ability to clearly distinguish signal patterns from these activities, confirming its potential for speech recognition applications. We further evaluated the device’s performance by analyzing five English vowel sounds (/ei/, /i:/, /ai/, /eu/, /ju:/), with the G-TENG showing excellent differentiation capability (Figure 4c). To quantify speech recognition accuracy, the volunteer continuously repeated the same word 60 times at a frequency of every 3 s to obtain a collection of waveforms for five words. And we extracted the mean, standard deviation, and peak width of the current signal of each word, fixing 80% of the training set and 20% of the test set. Using the K-nearest neighbor (KNN) algorithm for classification, we achieved an overall recognition accuracy of 96.56% (Figure 4e). To investigate practical application factors, we examined volume effects by recording the word “quiet” at different intensities (Figure 4f). The results showed consistent waveform patterns across volumes, with amplitude positively correlating to the sound level but no significant feature distortion, indicating stable performance regardless of volume variations. We further evaluated environmental noise resistance by comparing the word “hello” spoken in quiet (40 dB) and noisy (90 dB) conditions (Figure 4g). The G-TENG system maintained accurate voice feature extraction despite noise interference, while conventional microphone signals showed substantial background noise contamination. This comparative analysis highlights the G-TENG’s superior environmental noise immunity, supporting its practical utility in real-world settings.

## 4. Conclusions

In summary, we endowed the CS-PAM gel with a lower modulus by post-treatment with LiCl solution, and therefore, it can better adapt to the modulus of the skin and various deformations. The higher ionic conductivity due to ionic penetration gives it an even better output in the mode of bilayer. A G-TENG sensor was constructed using the gel as an electrode for monitoring physiological and acoustic signals. The obtained G-TENG exhibited high sensitivity and excellent reproducibility for accurate waveform recording of vibration signals from 0 to 1000 Hz. For waveforms of a single frequency, the detection error was below 0.20%. In addition, we used the G-TENG to record waveforms of different activities at the throat, demonstrating superior discrimination. Five words commonly used in daily life were collected, the corresponding dataset was built, and the average accuracy of recognizing the words was 96.56%. Moreover, the G-TENG is able to convey messages under different external conditions—both in quiet situations and noisy environments. These results highlight the great potential of G-TENG in the fields of self-powered wearable electronics, big data, and IoT. In the future, we will develop more efficient anti-freezing ionogels and sealing materials to improve various properties of the G-TENG and explore more applications in sensing.

## Data Availability

The original contributions presented in this study are included in the article. Further inquiries can be directed to the corresponding authors.
